# The complete mitochondrial genome of *Sinopodisma hengshanica* (Orthoptera: Acrididae) and its phylogenetic implication

**DOI:** 10.1080/23802359.2022.2060144

**Published:** 2022-04-04

**Authors:** Ran Li, Meng Jiao, Yujian Li, Lina Jiang

**Affiliations:** a School of Life Sciences, Qufu Normal University, Qufu, PR China; b Shandong Museum, Jinan, PR China

**Keywords:** *Sinopodisma hengshanica*, mitogenome, phylogeny, Acrididae, Orthoptera

## Abstract

The complete mitochondrial genome (mitogenome) of *Sinopodisma hengshanica* (Orthoptera: Acrididae) was firstly determined and analyzed in the present study. Assembled mitogenome sequence of *S. hengshanica* is 15,623 bp in size, containing 13 protein-coding genes (PCGs), two ribosomal RNA genes (rRNAs), 22 transfer RNA genes (tRNAs), as well as one A + T-rich region. Its gene component and arrangement are identical with other Acrididae species. The overall nucleotide composition is as follows: A (42.89%), G (10.34%), T (33.07%), and C (13.70%). All PCGs are initiated by typical ATN codons, and terminated with harboring complete stop codons TAA and TAG. Furthermore, phylogenetic trees were reconstructed based on 13 PCGs to validate the taxonomic status of *S. hengshanica*, exhibiting a close relationship with *Sinopodisma rostellocerca*.

The species of genus *Sinopodisma* Chang, 1940 belongs to the subfamily Melanoplinae, within the family Acrididae of the superfamily Acridoidea (Caelifera: Orthoptera). It currently includes 37 described species, which are mainly distributed in Eastern Asia (Yin et al. 2015). Mitochondrial genomes (mitogenomes) have been extensively used in researches of species identification, molecular phylogenetics and evolution in diverse insect taxa (Li et al. 2020). Hence, we sequenced and analyzed the complete mitogenome of *Sinopodisma hengshanica* Fu, 1998 to obtain the phylogenetic position and basic genetic information about this species.

Fresh specimens of *S. hengshanica* were collected from Changde of Hunan Province, China (29°2′39″N, 111°44′36″E). All samples were immediately preserved in absolute ethyl alcohol, and then stored at −20 °C. A specimen was deposited in the laboratory of School of Life Sciences, Qufu Normal University, Shandong, China (https://www.qfnu.edu.cn/, Lina Jiang, firstna@163.com) under the voucher number QF2021O. Total genomic DNA was extracted from the femoral muscles using a Wizard^®^ Genomic DNA Purification Kit (Promega, Madison, WI). The quality of DNA was examined by Nanodrop 2000 spectrophotometer (Thermo, Waltham, MA). The mitogenome sequence was amplified and sequenced using primer-walking strategy from both strands. Certain pairs of universal primers for locusts were used for polymerase chain reaction (PCR) amplification (Simon et al. 2006), and species-specific primers were designed to obtain overlapping segments of the whole sequence. Mitogenome was assembled by SeqMan program from DNASTAR (Burland 2000). Genome annotation was performed using MITOS under the code for invertebrate mitochondria (Bernt et al. 2013). The specimens were collected from meadow, no specific permissions were required for the locations. The species in our study is agricultural pest and is not included in the ‘List of Protected Animals in China’.

The whole mitogenome of *S. hengshanica* was determined to be 15,623 bp in length (GenBank accession no. MK352101), consisted of 13 typical protein-coding genes (PCGs), two ribosomal RNA genes (rRNAs), 22 transfer RNA genes (tRNAs), and one A + T-rich region. The orientation and gene order were in keeping with those of other known acridid species. The overall nucleotide composition of H-strand was biased toward 75.96% AT-content (A = 42.89%, T = 33.07%, G = 10.34%, and C = 13.70%). All 13 PCGs started with typical ATN codons (one with ATA, two with ATC, two with ATT, and nine with ATG), and ended with complete stop codons (two with TAG and 11 with TAA). The 16S and 12S genes were 1317 bp and 796 bp in size, respectively. Meanwhile, the mitogenome contained a typical set of 22 tRNAs, the size of which ranged from 64 bp to 71 bp.

To validate the new determined sequence and the taxonomic status of *S. hengshanica*, the phylogenetic trees were reconstructed with two methods: Maximum likelihood (ML) using RAxML 8.2.0 (Stamatakis 2014) and Bayesian inference (BI) using MrBayes 3.2.7a (Ronquist et al. 2012). All PCGs of 30 Melanoplinae species and three outgroups were used for phylogenetic analyses. As shown in Figure 1, phylogenetic trees using two inference methods yielded the same topology. Our phylogenetic analyses exhibited a close relationship between *S. hengshanica* and *S. rostellocerca* within the genus of *Sinopodisma*. In total, the new mitogenome obtained in this study provides essential data for species identification, and would give us a better understanding of the evolutionary and phylogenetic relationships among Acrididae.

**Figure 1.**
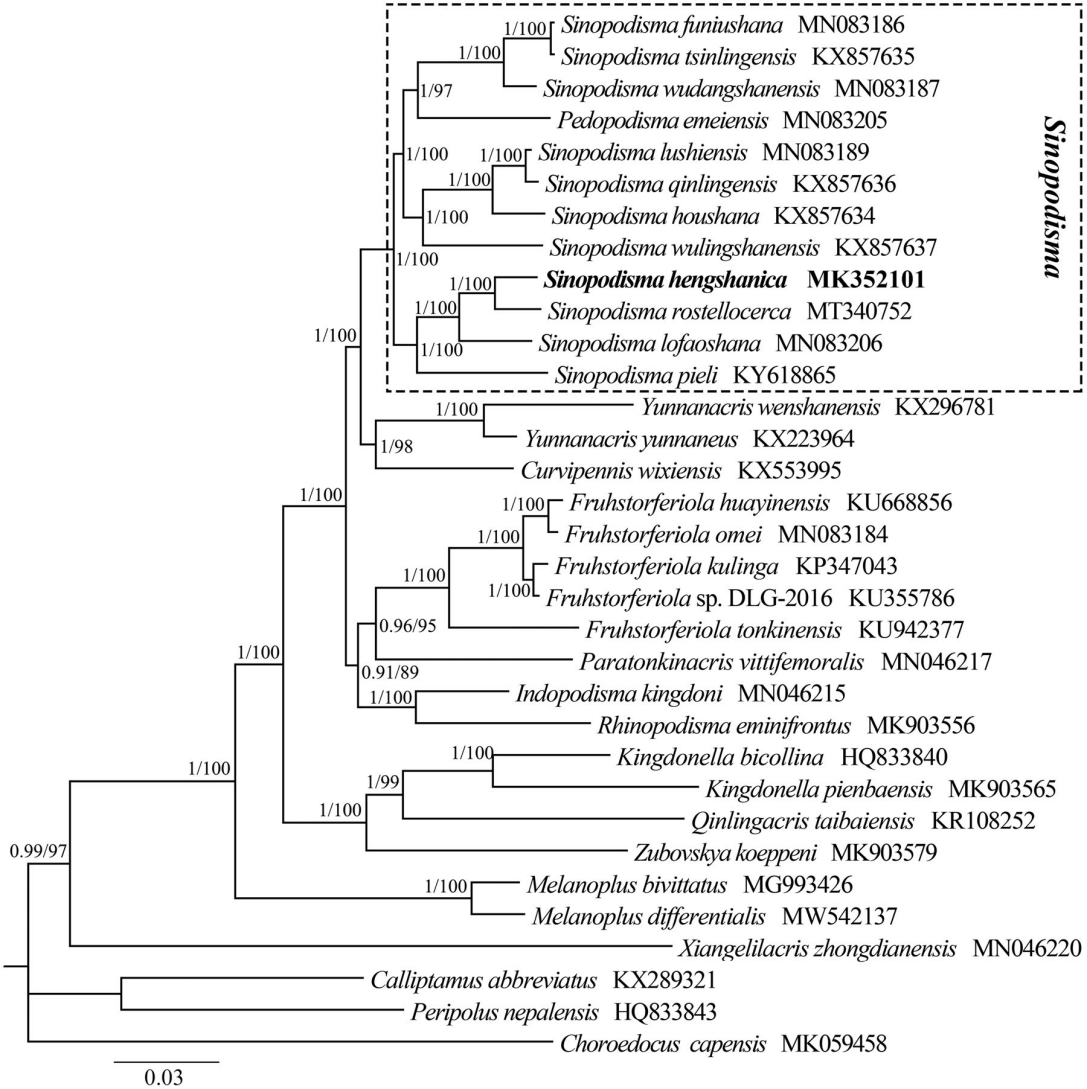
Phylogenetic tree obtained from ML and BI analysis based on 13 concatenated mitochondrial PCGs. Numbers on node are posterior probability (PP) and bootstrap value (BV).

## Data Availability

The genome sequence data that support the findings of this study are openly available in GenBank of NCBI at https://www.ncbi.nlm.nih.gov/ under the accession no. MK352101. Our mitogenome sequence was generated by Sanger sequencing in this study, so there is no BioProject, SRA, and BioSample accession numbers, and the genome sequence data have been released in GenBank of NCBI.
